# Toll-like receptor 2 is increased in neurons in Parkinson’s disease brain and may contribute to alpha-synuclein pathology

**DOI:** 10.1007/s00401-016-1648-8

**Published:** 2016-11-25

**Authors:** Nicolas Dzamko, Amanda Gysbers, Gayathri Perera, Anita Bahar, Amrita Shankar, Jianqun Gao, YuHong Fu, Glenda M. Halliday

**Affiliations:** 1 Neuroscience Research Australia, Barker Street, Randwick, NSW 2031 Australia; 2 School of Medical Sciences, University of NSW, Sydney, NSW 2052 Australia

**Keywords:** Parkinson’s disease, Toll-like receptor, α-Synuclein, Autophagy, Inflammation

## Abstract

Inflammation is likely a key contributor to the pathogenesis of Parkinson’s disease (PD), a progressively debilitating neurodegenerative disease that is accompanied by a pathological accumulation of the α-synuclein protein in a staged manner through the brain. What leads to the accumulation of α-synuclein in PD and how this relates to inflammatory pathways, however, is not entirely clear. Toll-like receptor (TLR) signaling is a major pathway mediating inflammation and, in particular, TLR2 is increasingly being implicated in PD. We have, therefore, examined the expression of TLR2 in postmortem brain tissue from PD patients and matched controls. We confirm that TLR2 is increased in PD brain, and find that levels of TLR2 correlate with the accumulation of pathological α-synuclein. TLR2 was expressed on neurons as well as microglia; however, the neuronal rather than glial expression of TLR2 was significantly increased in PD brain in accordance with disease staging, and TLR2 was strongly localized to α-synuclein positive Lewy bodies. In cell culture, activation of neuronal TLR2 induced an inflammatory response, including the secretion of inflammatory cytokines and microglial-activating chemokines, as well as the production of reactive oxygen species. Moreover, activation of neuronal TLR2 increased levels of endogenous α-synuclein protein, which was in turn associated with increased levels of the autophagy/lysosomal pathway marker p62. Finally, promoting autophagy with rapamycin or pharmacological inhibition of the TLR2 signaling pathway prevented the TLR2-mediated increase in α-synuclein in neuronal cell cultures. These results implicate neuronal TLR2 expression in human PD pathogenesis. In particular, the increased expression of TLR2 on neurons may provide new insight into disease pathogenesis and/or options for therapeutic intervention.

**Electronic supplementary material:**

The online version of this article (doi:10.1007/s00401-016-1648-8) contains supplementary material, which is available to authorized users.

## Introduction

Parkinson’s disease (PD) is a progressively debilitating neurodegenerative disorder. It is characterized by motor symptoms such as tremor, rigidity, bradykinesia and gait imbalance, as well as non-motor symptoms such as executive dysfunction deficits, sleep disturbances and depression [13]. Pathologically, PD is defined by the selective degeneration of dopamine-producing neurons in the substantia nigra pars compacta with concomitant accumulation of α-synuclein-rich proteinaceous neuronal inclusions, termed Lewy bodies, which propagate in a predicative or staged fashion through multiple brain regions [5, 16, 38].

Although exact causes of PD are unknown, accumulating evidence suggests an important role for inflammation in the pathogenesis of the disease. A number of genes that can either directly cause PD or contribute to its risk are implicated in inflammatory pathways [10, 12]. Inflammatory cytokines are increased in the brain, CSF and serum of PD patients [7, 17, 31] and activated microglia are prevalent in the most pathologically effected areas of the brains of patients with PD [26]. Despite such evidence, however, little is known about the pathways mediating inflammation and whether they are altered in brains of patients with PD.

One prominent inflammatory-regulating pathway is the toll-like receptor (TLR) pathway. TLRs mediate the recognition of exogenous conserved structures known as pathogen-associated molecular patterns (PAMPs) [18], and endogenous damage-associated molecular patterns (DAMPs) [33]. PAMPS derive from a range of microorganisms absent from the host such as viruses, bacteria, fungi and parasites, while DAMPs are released following cellular stress and from damaged tissues [41]. The consequences of TLR activation are well established for classical immune cells, such as macrophages and microglia, and include the production of pro-inflammatory cytokines such as tumor necrosis factor α and interleukin 1β, as well as the production of mitochondrial reactive oxygen species.

Recent reports have suggested that α-synuclein is a DAMP, capable of modulating inflammatory cytokine production in microglia [1, 3, 21, 39, 47]. As well as forming insoluble Lewy body aggregates in PD brain, α-synuclein also forms monomers, oligomers, fibrils and other conformations [44]. The exact contribution of different α-synuclein conformations to TLR activation is currently unclear; however, it has been demonstrated that α-synuclein can at least activate the TLR2 receptor on microglia and promote inflammatory cytokine production [9, 15, 19]. Moreover, α-synuclein can increase the expression of TLRs [4], suggesting that brain regions that accumulate α-synuclein in PD may have increased TLR2 protein. We, therefore, tested this hypothesis and indeed found a strong association of TLR2 with α-synuclein, both of which were increased in the affected brain regions in patients with PD. Intriguingly, the increased TLR2 protein levels were associated with neurons, rather than microglia, in PD brain and TLR2 was a robust component of Lewy bodies. Experiments activating neuronal TLR2 resulted in the production of inflammatory cytokines, and increased oxidative stress and accumulation of endogenous α-synuclein protein in cell culture models, including primary human neurons derived from induced pluripotent stem cells. As it is neurons rather than glia that accumulate α-synuclein in PD, these results suggest that TLR2 may be of central importance in disease pathogenesis.

## Materials and methods

### Cases

Brain tissues from longitudinally followed, autopsy-confirmed subjects with sporadic PD (*n* = 17) and age- and postmortem delay-matched clinical and neuropathological controls (*n* = 10) (Table 1) were obtained from the Sydney Brain Bank following study approval and University of New South Wales human ethics approval (#HC14046). All PD cases met the UK Brain Bank Clinical Criteria for diagnosis and had no other neurodegenerative conditions. The cases analyzed have been previously used to assess lysosomal proteins, lipids, and α-synuclein levels in the affected brain regions [29].

**Table 1:** Demographic details for Parkinson’s disease (PD) and control cohorts

	Control (*n* = 10)	All PD (*n* = 17)	Early stage PD (*n* = 6)	Late stage PD (*n* = 11)
Sex (M:F)	5:5	13:4	5:1	8:3
Age at death (years)*	74.7 ± 2.9 (60–88)	78.4 ± 1.3 (69–88)	78.8 ± 2.7 (71–88)	78.2 ± 1.4 (69–85)
Postmortem delay (h)*	18.2 ± 3.1 (7–35)	14.8 ± 2.6 (3–42)	12.0 ± 2.9 (3–23)	16.4 ± 3.9 (3–42)
Disease duration (years)*	–	14.8 ± 1.6 (7–36)	14.3 ± 1.8 (8–20)	15.0 ± 2.4 (7–36)
Braak Lewy stage (/6)	0	4.9 ± 0.2 6IV:7V:4VI	4.0 ± 0 6IV	5.4 ± 0.2 7V:4VI
Braak neuritic stage (/6)^#^	0.6 ± 0.3 (0–2)	1.2 ± 0.3 (0–4)	1.2 ± 0.3 (0–2)	1.3 ± 0.4 (0–4)
CERAD plaque score (/3)^#^	0.3 ± 0.2 (0–2)	0.9 ± 0.2 (0–3)	0.7 ± 0.3 (0–2)	1.1 ± 0.3 (0–3)

Values are given as mean ± standard error and range for age at death, postmortem delay, disease duration, Parkinson’s disease severity (Braak Lewy stage) and Alzheimer’s disease severity (CERAD plaque score and Braak neuritic stage)

* Not significantly different between groups (*P* > 0.3), ^#^ PD cases and controls do not meet diagnostic criteria for Alzheimer’s disease with CERAD scores and Braak neuritic stages not significantly different between groups (*P* = 0.16 and *P* = 0.44, respectively)

### Protein extraction from frozen brain tissue

Tris-buffered saline (TBS) and SDS-soluble proteins were serially extracted from 250 mg of fresh-frozen brain tissue from the anterior cingulate cortex and occipital cortex as previously described [29]. Briefly, tissue was mechanically homogenized in ten volumes of TBS homogenization buffer (50 mM Tris, 125 mM NaCl, pH 7.4, 5 mM EDTA, 0.02% sodium azide) containing protease inhibitor cocktail (Roche), followed by centrifugation at 100,000×*g* for 2 h at 4 °C, with supernatant collected as the TBS-soluble fraction containing cytosolic proteins. The pellet was resuspended in SDS solubilization buffer (TBS homogenization buffer containing 5% SDS), sonicated (2 × 10 s bursts) and centrifuged at 100,000×*g* for 30 min at 25 °C, with supernatant collected as the SDS-soluble fraction containing membrane-associated proteins. Protein concentration of all fractions was measured using a bicinchoninic acid assay (Pierce BCA Protein Assay Kit, Thermo Scientific), according to the manufacturer’s instructions. Samples were stored at −80 °C until use.

### Immunoblotting

Up to 30 μg of protein lysate was heated with sample buffer (2% SDS, 20% glycerol, 2.5% bromophenol blue, 12.5 mM Tris–HCl, pH 6.8, 5% 2-mercaptoethanol) and separated by reducing SDS-PAGE before transfer to nitrocellulose membrane (BioRad). Membranes were fixed in 0.4% paraformaldehyde followed by blocking in 5% skim milk dissolved in 1 × TBS-T (0.87% NaCl, 0.01 M Tris, pH 7.4, with 0.1% Tween20). Membranes were then cut into strips based on molecular weight markers and incubated overnight in primary antibodies prior to protein detection using either horseradish peroxidase-conjugated secondary antibodies (Biorad) with enhanced chemiluminescence (Amersham ECL Plus Western Blot Detection System, GE Healthcare) or Alexa Fluor-conjugated secondary antibodies (Life Technologies). Primary antibodies for immunoblotting were rabbit monoclonal TLR2 (Abcam, 1:500 dilution), rabbit monoclonal TLR1 (Abcam 1:1000 dilution) mouse monoclonal NeuN (Millipore, 1:250 dilution), mouse monoclonal α-synuclein (BD Biosciences, 1:3000 dilution), phospho-α-synuclein (P-129, Abcam, 1:1000 dilution), MYD88, phospho-NFκBp105 (Ser933), phospho-p38 MAPK (Thr180/Tyr182), p62/SQSTM1 and Beclin1 (all Cell Signaling Technology, 1:1000 dilution), HLA-DR (Dako, 1:5000 dilution), LC-3 (Novus, 1:100 dilution) and goat polyclonal Iba1 (Abcam, 1:1000 dilution) with β-actin (Abcam, 1:50,000 dilution) used as a protein loading control. For immunoblotting of Ser-129 phosphorylated α-synuclein, membranes were fixed with 4% PFA and 0.1% glutaraldehyde prior to blocking as described [36]. A Biorad Chemidoc MP system was used to capture images and the relative levels of each protein of interest were analyzed using Image J software (US National Institutes of Health). The intensity of each protein band was quantified and expressed as arbitrary units standardized to β-actin.

### Immunofluorescent labeling using human tissue

Immunohistochemistry was performed with 10 μm formalin-fixed paraffin-embedded tissue sections from the substantia nigra and anterior cingulate cortex of 50% of the cases used for immunoblotting (9 PD and 5 matched control cases randomly chosen). Briefly, sections were deparaffinized in xylene and rehydrated in graded ethanols. Antigen retrieval was performed using 99% formic acid for α-synuclein immunohistochemistry, and boiling with citrate buffer (pH 6.0) for all other antibodies, after which sections were cooled to room temperature. Throughout the protocol, all washes were three times with 0.1 M Tris buffer. For immunofluorescent double labeling, sections were blocked with 10% horse serum in TBS buffer containing 0.5% triton-X-100 and incubated overnight with primary antibody pairs: anti-α-synuclein (1:200 dilution) and Abcam anti-TLR2 (1:100 dilution) or anti-Iba1 (1:400 dilution) and anti-TLR2 or anti-NeuN (1:200 dilution) and anti-TLR2 or anti-tyrosine hydroxylase (Sigma, 1:400 dilution) and anti-TLR2. Alexa fluor-labeled secondary antibodies (Life Technologies) diluted 1:500 in 0.1 M Tris buffer were then used. Auto-fluorescence was reduced by incubating in autofluorescence eliminator buffer (Millipore) following the manufacturer’s instructions. Slides were then stained with DAPI (Sigma) and coverslipped using Vectashield mounting medium (Vector Laboratories). Images were visualized using a Nikon Confocal Microscope ECLIPSE 90i and captured using EZ C3.80 software. At least ten images from each slide of the anterior cingulate cortex were used to quantify (1) Iba1-immunoreactive microglia expressing TLR2 immunoreactivity, (2) all DAPI-labeled neuronal nuclei expressing TLR2 immunoreactivity, and (3) α-synuclein-immunopositive Lewy bodies colocalizing TLR2 immunoreactivity. Neurons were identified by their nucleus being wider and fainter with a bright nucleolus compared with the nuclei of surrounding glial cells in the DAPI stain. Quantitation was performed using 590 microglia (average of 42 ± 12 per case), 1483 neurons (average of 106 ± 70 per case), and 117 Lewy bodies (average 23 ± 6 per case that contained Lewy bodies in the cingulate cortex). To assess interrater variation in the cell counts, 16 ± 6 images from each combination of double labeling were used. There was <5% variation in counts made by two researchers, with a correlation between counts of 0.96.

### Immunoperoxidase staining using human tissue

For immunoperoxidase staining, sections were incubated with either rabbit monoclonal anti-TLR2 from abcam, or goat polyclonal anti-TLR2 antibody from R&D systems, as outlined in the supplementary methods. Absorption controls were also performed for both TLR2 antibodies to demonstrate the antibody binding specificity. The antigen to antibody mixture was made at a working dilution of 10:1 (molar ratio) and was pre-incubated overnight at 4 °C. The pre-absorbed antibodies were then be incubated with tissue sections in place of the primary antibodies with the same immunoperoxidase staining protocols for the respective TLR2 antibody.

### Tissue culture experiments with SHSY5Y cells

Human neuroblastoma SHSY5Y cells were cultured in Dulbecco’s modified Eagle medium/Hams F12 supplemented with 10% low endotoxin fetal bovine serum and 1 × penicillin/streptomycin solution (all from Gibco, Life Technologies). Cells were differentiated for 7 days in the same media except with 1% fetal bovine serum and 10 μM retinoic acid (Sigma). Cells were treated with TLR agonists (all purchased from Invivogen) dissolved in endotoxin-free water and/or small molecule inhibitors (Calbiochem, Cayman Chemicals or Sigma) dissolved in DMSO at indicated concentrations for indicated time points. For cytokine ELISA assays, tissue culture media were removed and snap-frozen in liquid nitrogen and stored at −80 °C until analysis. For gene expression analysis, cells were lysed according to the manufacturer’s instructions (ReliaPrep, miniprep systems, Promega) and stored at −80 °C. For immunoblot analysis, cells were lysed either in buffer containing 50 mM Tris HCl pH 7.5, 1 mM EGTA, 1 mM EDTA, 1 mM sodium orthovanadate, 50 mM sodium fluoride, 5 mM sodium pyrophosphate, 0.27 M sucrose, 1 mM benzamadine, 1 mM phenylmethylsulfonyl fluoride and 1% (v/v) Triton X-100, or straight into 1 × LDS sample buffer (Life Technologies). For the former, lysates were clarified by centrifugation at 13,000×*g* for 20 min and protein concentrations were measured by bicinchoninic acid assay as mentioned above. Reactive oxygen species were measured using a 2′,7′-dichlorofluorescin diacetate (DCFDA) assay (Abcam) following the manufacturer’s instructions. Briefly, 25,000 cells were seeded into 96 well clear bottom microplates and differentiated for 7 d before treatment for 4 h with 1 μg/ml PAM3CSK4. The included 55 mM tert butyl hydrogen peroxide was used as a positive control. Median fluorescence intensity was measured using a fluorescence microplate reader (Polarstar Omega, BMG). Apoptosis and cell viability were assessed by Annexin V/propidium iodide staining following the manufacturer’s instructions (Annexin V-FITC kit from Miltenyi Biotech). Median fluorescence intensity was measured using a FACS Canto cell analyzer (BD Biosciences) with at least 20,000 events captured. Flow cytometry data were acquired with FACS Diva software (BD Biosciences) and analyzed using FlowJo software (Tree Star).

### Immunocytochemistry

Coverslips with adherent cells were washed in 1 × PBS and fixed in 4% paraformaldehyde for 15 min at room temperature. Coverslips were washed again in 1 × PBS after fixing and permeabilized with 0.3% Triton X-100 for 15 min. Cells were then blocked in 3% BSA for 1 h and incubated overnight at 4 °C in primary antibody at a dilution of 1:200 in 3% BSA. After incubation, cells were washed 3 × 5 min in 1 × PBS and incubated with Alexa Fluor secondary antibodies (Abcam, 1:300 dilution) in 3% BSA for 1 h at room temperature in the dark. Cells were washed again for 3 × 5 min in 1 × PBS with DAPI added to the last wash at a concentration of 1:10,000. Coverslips were mounted face down using fluorescent mounting medium (Dako) and allowed to dry in the dark for 1 h before visualizing. Images were captured on a confocal microscope (Nikon) using NIS elements AR software. Images were obtained in each channel at 40× magnification for analysis. Intensity settings were kept constant for all images, which were analyzed using Image J 1.49v (NIH). The threshold tool on Image J was used to highlight all stained areas within the cells and the intensity of each highlighted particle was obtained using the analyze particles tool. Once selected, the threshold was kept constant for all images. The cell counter plugin was used to count the number of DAPI positive cells in the image and a total of 400–500 cells were counted for each condition. Cells falling on the edges of the image were excluded from the analysis.

### Induced pluripotent stem cells

Human fibroblasts from a neurologically normal subject were obtained from the NINDS genetic repository at Coriell (#ND38530). Briefly, primary fibroblasts at passage 4 were reprogrammed to induced pluripotent stem cells (IPSCs) using the EPi5 reprogramming kit (Life Technologies) as per the manufacturer’s instructions. Electroporation of fibroblasts was performed with the Neon transfection system (Life technologies) and colonies with the morphology of IPSCs were selected ~30 days post-electroporation. IPSCs were maintained on geltrex (Life Technologies)-coated dishes and stained for both pluripotency (Pluripotent stem cell marker immunocytochemistry kit, Life Technologies) and tri-lineage differentiation potential (Human pluripotent stem cell functional identification kit, R&D Systems) as per the manufacturer’s instructions. Neural progenitor cells were then derived from IPSCs using the PSC neural induction kit (Life Technologies) as per the manufacturer’s instructions. Differentiation of neural progenitor cells was confirmed by immunocytochemistry for nestin and paired box protein 6 (PAX6). Only cell lines confirmed >95% positive for both markers were used. Neural stem cells were maintained in neural expansion medium comprising 50% RPMI media (Life Technologies) and 50% neurobasal medium (Life Technologies), supplemented with neural induction supplement at 1/50 dilution and penicillin–streptomycin at 1/100 dilution. Media were changed every second day and neural stem cells were passaged in the presence of ROCK inhibitor Y27632 (Cayman Chemicals) at a final concentration of 5 µM. For all experiments described, neural stem cells were used at a passage <8. Images were captured on a confocal microscope (Nikon) using NIS Elements software (Nikon). For the directed differentiation to neurons, neural progenitor cells were plated at a density of 2.5 × 10^4^ cells per ml on coverslips coated with poly-l-ornithine (Sigma) and laminin (10 μg/ml, Life Technologies). Cells were cultured in neurobasal media containing 2% B27 supplement and 2 mM glutamax (all Life Technologies). For the first 3 days, media were changed daily. After this, half of the media were replaced every second day and it was supplemented with 1 μg/ml PAM3CSK4. In total, cells were differentiated for 10 days with 7 days of PAM3CSK4 treatment. At this stage, neurons no longer expressed nestin, but now expressed the neuronal markers MAP2 and TUJ1.

### qRT-PCR and ELISA

RNA was extracted using the ReliaPrep miniprep system (Promega) following the manufacturer’s instructions. RNA was reverse transcribed to cDNA using the iScript cDNA synthesis kit (Biorad). qRT-PCR was performed on a realplex thermocycler (Eppendorf) using sybr green-based chemistry (Biorad). All primer sequences were obtained from qPrimer depot [8] and synthesized by Sigma. Gene expression was quantified using the comparative Ct method [24] with glyceraldehyde-3-phosphate dehydrogenase (GAPDH) as the housekeeping gene. Melt-curve analysis was performed to ensure that a single product was amplified. Cytokine ELISA assays were performed using Bioplex xMAP assays (Biorad) following the manufacturer’s protocol with plates read on a Magpix instrument (Luminex).

### Statistical analysis

All statistical analyses for human tissue were performed using SPSS Statistics software (IBM, Chicago, IL, USA) and statistical significance set at *p* < 0.05. Group demographic statistics have been previously reported [29] with cases well matched for age, sex and postmortem delay (Table 1), and univariate statistical analysis covarying for neuron loss (NeuN levels) shows that the relative levels of SDS-soluble α-synuclein protein increased in PD [29]. There was also a positive Spearman’s correlation between the levels of SDS-soluble α-synuclein protein and Braak Lewy body stage of Parkinson’s disease for these cases [29].

To determine TLR2 levels and location changes in PD, two multivariate analyses were performed, the first on the quantitated immunoblot data to identify any changes in TLR2 levels in association with α-synuclein levels (covarying for age, postmortem delay and levels of NeuN, Iba1 and HLA-DR), and the second on the quantitated immunohistochemistry data to identify any changes in the numbers of TLR2-immunopositive microglia, neurons and Lewy bodies (covarying for age, postmortem delay and Braak neuritic stage). Non-parametric Spearman’s correlations were used to identify related variables.

Statistical analysis of data obtained from tissue culture experiments was performed using Graphpad software (Prism). Students *t* test was used for comparisons between two groups and one-way ANOVA with Tukey’s post hoc test was used for comparisons between multiple groups. Significance was accepted at *p* < 0.05 and data are presented as mean ± standard error of the mean.

## Results

### TLR2 is increased in the affected brain regions in PD and correlates with pathological α-synuclein levels

**Fig. 1:**
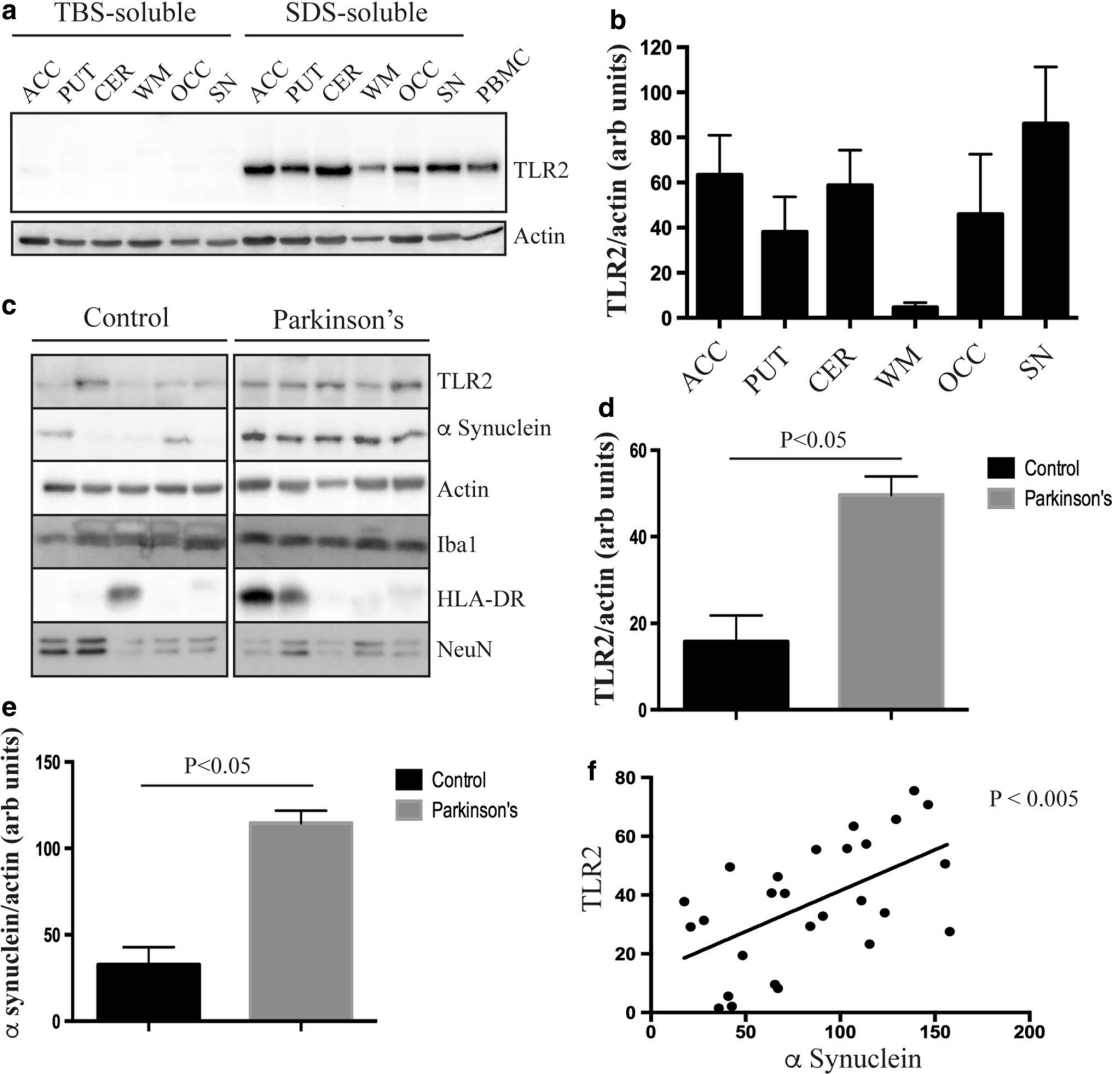
Toll-like receptor 2 protein is increased in Parkinson’s disease brain. **a** Immunoblot analysis of TLR2 from different regions of healthy brain following sequential extraction of proteins in tris-buffered saline (TBS buffer) followed by SDS buffer. *ACC* anterior cingulate cortex, *PUT* putamen, *CER* cerebellum, *WM* white matter, *OCC* occipital cortex, *SN* substantia nigra. Human peripheral blood mononuclear cells (PBMCs) were used as a positive control for TLR2 expression. β-Actin was used as a loading control. Immunoblot images are representative of *n* = 3 cases. **b** Quantification of relative TLR2 expression in different human brain regions after normalization to β-actin. Data are mean ± SEM, *n* = 3. **c** Representative immunoblot images from control and Parkinson’s disease (PD) anterior cingulate cortex. Quantification of TLR2 **(d)** and total α-synuclein **(e)** protein in control and PD brain tissue after normalization to β-actin. Data are mean ± SEM, *n* = 10 controls and 17 PD cases. Multivariate analysis was used to determine a significant difference at *p* < 0.05. **f** A significant correlation was observed between TLR2 and α-synuclein proteins for all cases (*n* = 27)

To determine the regional expression and relative solubility of TLR2 in postmortem brain tissue, sequential TBS- and SDS-soluble protein lysates from the anterior cingulate cortex, putamen, cerebellum, substantia nigra, occipital cortex and white matter were immunoblotted for TLR2. Consistent with TLR2 being predominantly membrane associated, the protein was detected only in the SDS-soluble fraction (Fig. 1a). Moreover, for the control subjects chosen, the regional expression of TLR2 appeared mostly uniform in gray matter regions, with reduced levels seen in white matter (Fig. 1a, b). Importantly, TLR2 was expressed in regions pathologically affected by PD (i.e., substantia nigra, anterior cingulate cortex and putamen).

To assess the effect of PD on TLR2 expression, SDS-soluble fractions from the anterior cingulate cortex were immunoblotted for TLR2, along with α-synuclein to assess PD pathology, and markers for specific brain cell types (NeuN for neurons, Iba1 (combined data from both the TBS and SDS-soluble fractions) and HLA-DR for microglia and activated microglia, respectively). The anterior cingulate cortex was chosen on the basis of Braak staging, in which we, and others [29], have shown that there is an increase in α-synuclein protein levels in this region early in PD (Braak stage IV) prior to the deposition of Lewy pathology and in the absence of neuronal loss. We did not quantitatively assess the substantia nigra because of the substantial loss of neurons in this region that would distort any data. Representative immunoblots from the SDS-soluble fraction of the anterior cingulate cortex are shown in Fig. 1c, with un-cropped images available as supplementary data (Supplementary Figure 1). As detailed in the methods, multivariate analysis of the quantitated immunoblot data was used to identify any changes in TLR2 levels, revealing a significant increase in TLR2 protein in the PD group (Fig. 1d). Age (*p* = 0.44) and postmortem delay (*p* = 0.47) had no effect on the protein levels of TLR2. Neither did levels of NeuN (*p* = 0.43), Iba1 (*p* = 0.49) or HLA-DR (*p* = 0.59) suggesting that the increased expression of TLR2 with PD was not due to differences in brain cell types, such as an increase in microglia. Spearman correlation analysis showed that a significant relationship between increasing SDS-soluble α-synuclein (Fig. 1e shows group comparisons, also shown previously [29]) and TLR2 levels (Fig. 1f) indicating that TLR2 protein is increased in PD brain in association with pathological α-synuclein. In contrast, we did not find an increase in either TLR2 or α-synuclein in the occipital cortex (Supplementary Figure 2), a region unaffected by Lewy pathology in PD.

### TLR2 is expressed on neurons as well as microglia

**Fig. 2:**
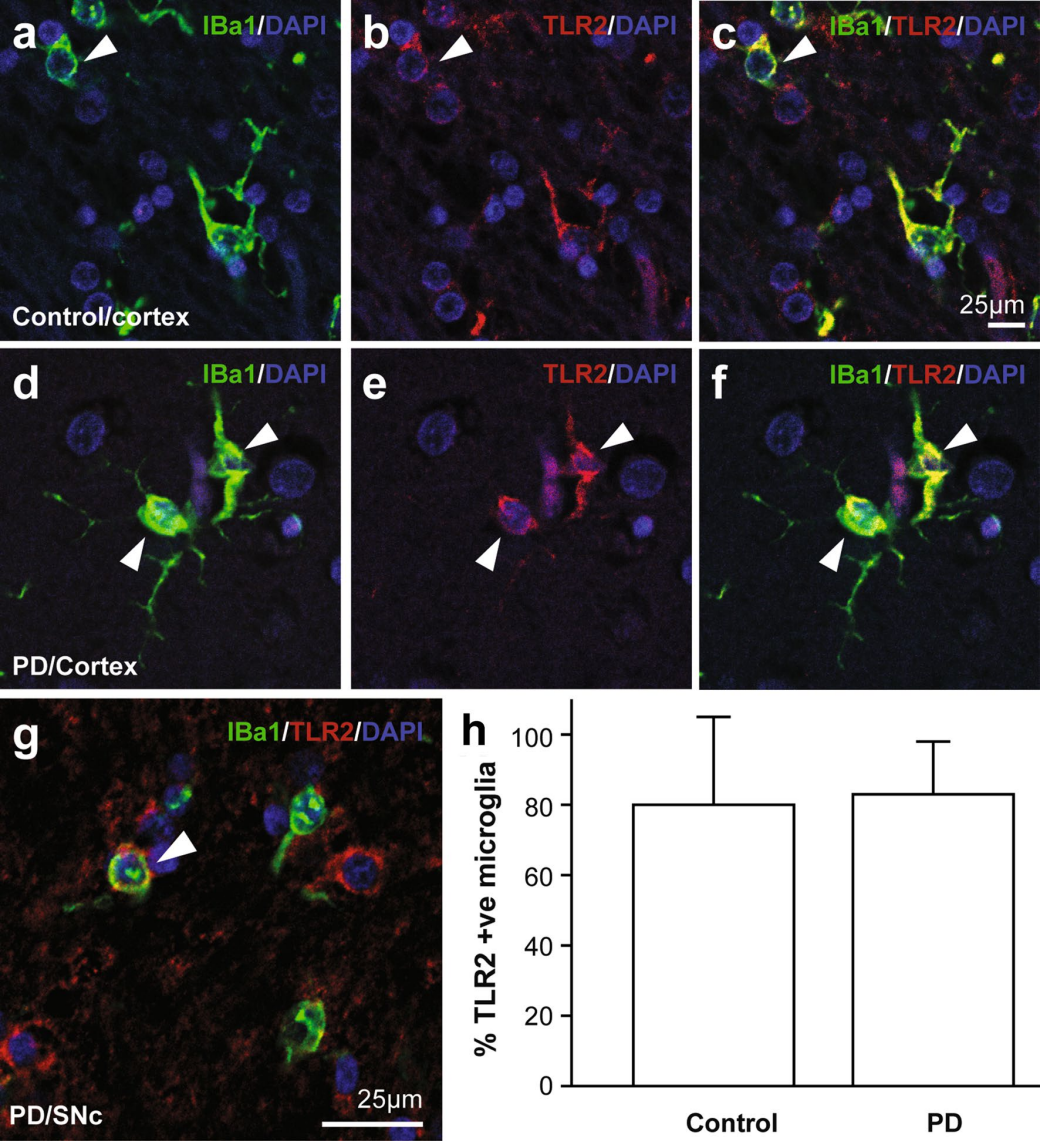
Toll-like receptor 2 protein is expressed by microglia in Parkinson’s disease brain. Double-fluorescence labeling of Iba1 positive microglia (*green*) and TLR2 (*red*) from control (**a**–**c**) and Parkinson’s disease (PD) (**d**–**f**) anterior cingulate cortex, and PD substantia nigra pars compacta (**g**). **h** Percentage of Iba1 positive microglia that co-localize TLR2 in control and PD anterior cingulate cortex. Data are mean ± SEM. A total of 590 microglia were assessed from 5 control and 9 PD cases

**Fig. 3:**
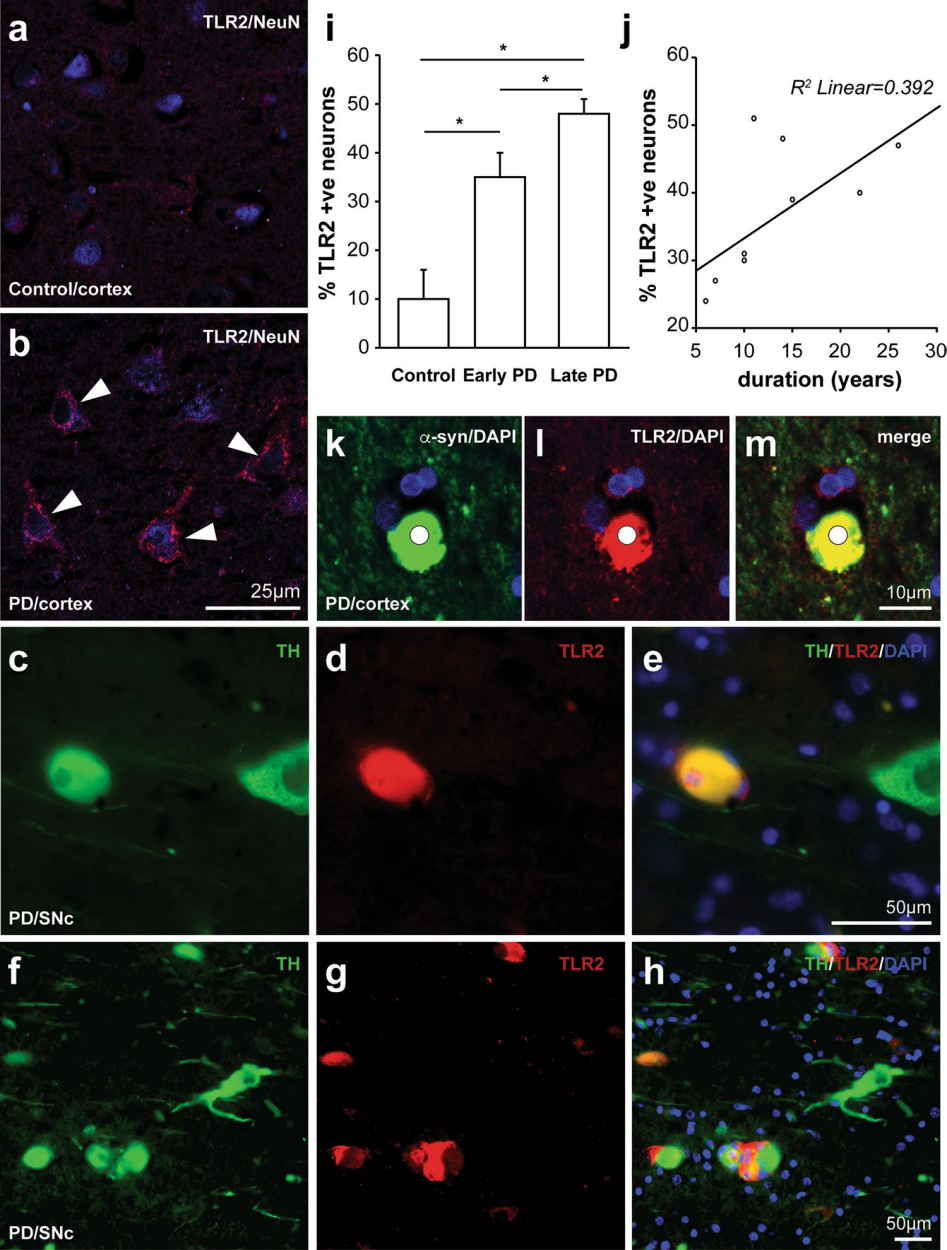
Increased toll-like receptor 2 protein is expressed by neurons in Parkinson’s disease brain. Fluorescence labeling of TLR2 (*red*) and NeuN (*blue*) in neurons from control (**a**) and Parkinson’s disease (PD) (**b**) anterior cingulate cortex, and TLR2 (*red*) and tyrosine hydroxylase (*green*) dopaminergic neurons in the substantia nigra pars compacta of two different PD patients (**c**–**h**). TLR2-positive dopaminergic neurons have overlapping yellow fluorescence (**e**, **h**). **i** Percentage of neurons co-localizing TLR2 in control, early and late stage PD. Data are mean ± SEM. A total of 1483 neurons were assessed from 5 control and 9 PD cases. *Asterisk* indicates *P* < 0.05. **j** Correlation between the percentage of neurons co-localizing TLR2 and PD duration (*n* = 9). **k**–**m** Double-fluorescence labeling of alpha-synuclein (*green*) with TLR (*red*) shows significant co-localization (*yellow*) in Lewy bodies (**m**)

The expression of TLR2 in PD brain was further assessed by immunohistochemistry (Figs. 2, 3, Supplementary Figure 3). In both the anterior cingulate cortex and substantia nigra, as expected, microglia robustly co-localized TLR2 immunoreactivity in both control and PD brain (Fig. 2a–g). To quantify the number of microglia expressing TLR2, counts of co-localized TLR2 and Iba1 immunoreactivity in microglia were performed. This analysis revealed that the majority of Iba1 positive microglia in both control and PD brain were also TLR2-immunopositive (80 ± 25% of control microglia, 83 ± 15% of PD microglia, *F* = 0.007, *p* = 0.94) (Fig. 2h). Age (*p* = 0.88), postmortem delay (*p* = 0.47) and Braak neuritic stage (*p* = 0.68) had no effect on the pattern of microglia TLR2 immunoreactivity.

In addition to microglia expressing TLR2, we observed TLR2-immunostaining on cells with the morphology of neurons that also stained positive for the neural marker NeuN. Compared to neurons from control brain (Fig. 3a), TLR2 immunoreactivity was much higher on neurons from PD brain in both the anterior cingulate cortex (Fig. 3b) and the substantia nigra (Fig. 3c–h). Immunoperoxidase staining using two different TLR2 antibodies also demonstrated an increased expression of TLR2 on neurons in the anterior cingulate of PD brain, with preabsorption controls confirming antibody specificity (Supplementary Figure 3). Quantification of the fluorescence images in the anterior cingulate cortex showed that relatively few neurons in controls were immunopositive for TLR2 (3 ± 6%), while this number was significantly increased in PD (47 ± 4%, *F* = 21.5, *p* = 0.001). Again, postmortem delay (*p* = 0.47) and Braak neuritic stage (*p* = 0.18) had no effect on the pattern of neuronal TLR2 immunoreactivity, but increasing age was associated with increasing neuronal TLR2 immunoreactivity (*p* = 0.04). These factors were covaried in the analysis and changes in early versus late stages of PD assessed with older cases only (*N* = 5 controls, *N* = 4 early stage disease, *N* = 5 late stage disease, *F* = 0.81, *p* = 0.47). Assessment of the changes with disease stage revealed increasing TLR2-immunopositivity in neurons with increasing stage (10 ± 6% of control neurons, 35 ± 5% of early stage neurons, 48 ± 3% of late stage neurons, *F* = 19.8, *p* = 0.001, post hoc Bonferroni-corrected *p* < 0.04 for all paired comparisons) (Fig. 2i). Spearman rank correlations confirmed a strong association between increasing disease stage and increasing TLR2-immunoreactivity in neurons (*ρ* = 0.87, *p* = 0.003). In the cases with PD, a correlation between longer disease durations and increasing TLR2-immunoreactivity in neurons (*ρ* = 0.73, *p* = 0.03) was also observed (Fig. 2j). In the substantia nigra, the increase in TLR2 immunoreactivity was observed in the remaining tyrosine hydroxylase expressing neurons in PD brain (Fig. 3), but due to the neuronal loss in this region we did not further quantify expression. These data show robust expression of TLR2 protein in neurons in patients with PD that increases with disease duration and the progression of α-synuclein pathology.

### TLR2 is a component of Lewy bodies

It was apparent from the analysis of TLR2 immunoreactivity in PD brain that Lewy bodies were also robustly TLR2-immunopositive (note controls did not have Lewy bodies). To quantify the expression of TLR2 in Lewy bodies, double labeling immunofluorescence was employed for TLR2 and α-synuclein (Fig. 2k–m), again using tissue from the anterior cingulate cortex. Quantification showed that most (92 ± 10%) α-synuclein-immunopositive Lewy bodies colocalized TLR2-immunoreactivity. As may be expected with this level of colocalization, there was no correlation with any of the covariates (*p* > 0.4) and also no correlation with disease duration (*p* = 0.78).

### Activation of neuronal TLR2 induces an inflammatory response

**Fig. 4:**
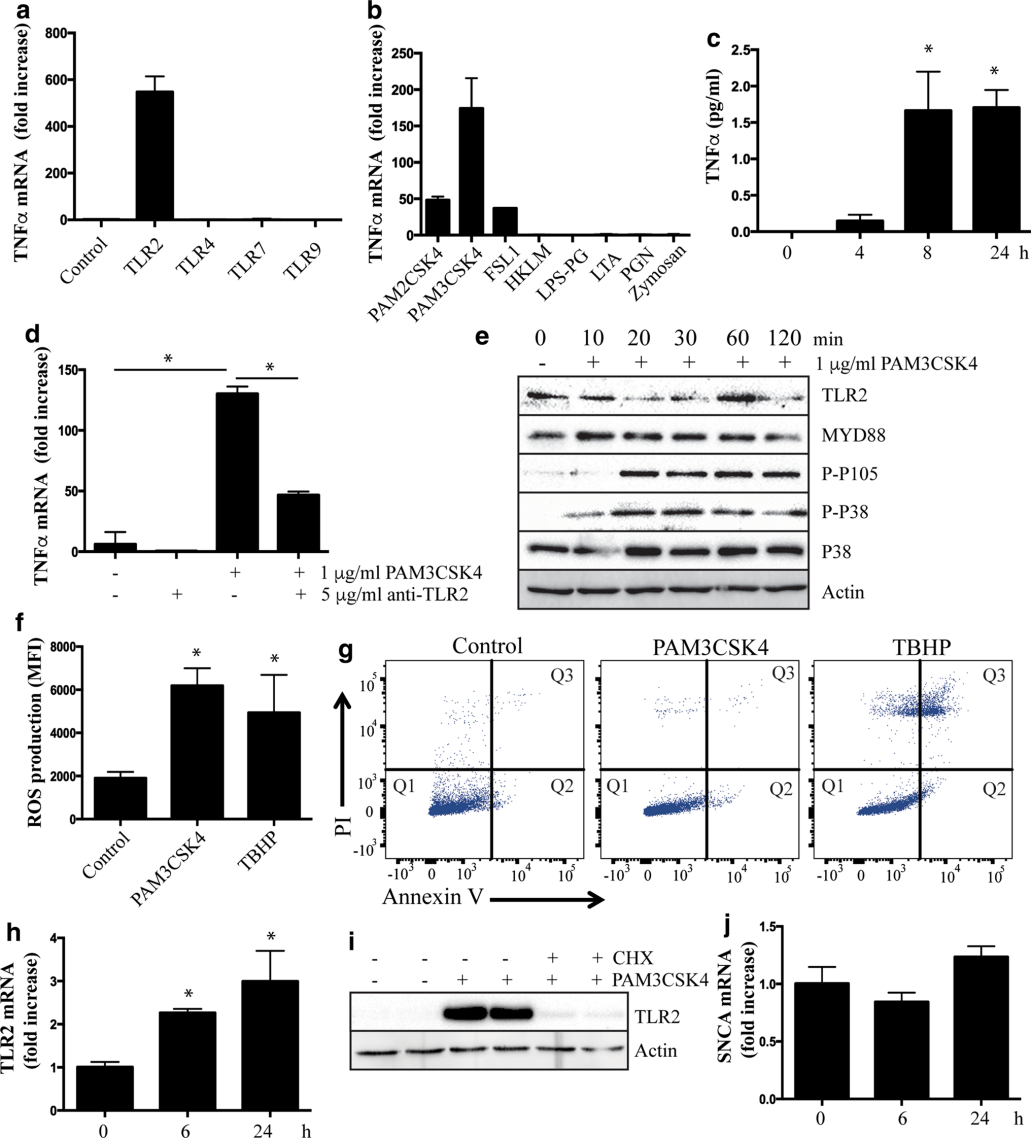
Activation of neuronal toll-like receptor 2 induces an inflammatory response. **a** Retinoic acid-differentiated SHSY5Y cells were treated with agonists of MYD88-depedent TLR signaling for 6 h before RNA was extracted for measurement of TNFα mRNA expression by qRT-PCR (TLR2 agonist = 1 μg/ml PAM3CSK4, TLR4 agonist = 1 μg/ml lipopolysaccharide, TLR7 agonist = 1 μg/ml CLO97 and TLR9 agonist = 2 μM ODN 2336). TNFα mRNA was not detected in untreated samples, or samples treated with agonists other than TLR2. TNFα crossed the threshold at ~cycle 30, with GAPDH at ~cycle 20. **b** TNFα mRNA expression was measured by qRT-PCR following treatment of differentiated SHSY5Y cells with a panel of TLR2 agonists for 6 h. PAM2CSK4 and PAM3CSK4 are di- and tri-acylated bacterial lipopeptides, respectively, FSL1 is a synthetic lipoprotein derived from *Mycoplasma salivarium*, HKLM is heat killed *Listeria monocytogenes*, LPS-PG is lipopolysaccharide from *Porphyomonas gingivalis*, LTA is lipoteichoic acid, PGN is peptidoglycan and Zymosan is a yeast cell wall component. **c** ELISA assay of TNFα protein secreted into tissue culture by differentiated SHSY5Y cells treated with 1 μg/ml PAM3CSK4 over a 24 h time course. **d** PAM3CSK4-induced expression of TNFα mRNA at 6 h is blunted by co-treatment with a TLR2 neutralizing antibody. **e** Differentiated SHSY5Y cells were treated with PAM3CSK4 over a 2 h time course and lysates generated for immunoblot of phosphorylated NFκB P105 subunit and phosphorylated P38 MAPK. **f** Reactive oxygen species measured by DCFDA assay following 24 h treatment of differentiated SHSY5Y cells with 1 μg/ml PAM3CSK4. **g** Flow cytometry scatter plots of annexin V and propidium iodide median fluorescence intensity in SHSY5Y cells treated with or without 1 μg/ml PAM3CSK4 or 50 μM tert-butyl H_2_O_2_ (TBHP) for 24 h. **h** mRNA expression of *TLR2* in differentiated SHSY5Y cells treated over 24 h with 1 μg/ml PAM3CSK4. **i** Increased expression of TLR2 protein following 12 h of 1 μg/ml PAM3CSK4 is blocked by 10 μg/ml of cyclohexamide. **j** mRNA expression of *SNCA* in differentiated SHSY5Y cells treated over 24 h with 1 μg/ml PAM3CSK4. For all graphs, data are presented as mean ± SEM with *n* = at least 6. All figures are representative of at least three independent experiments. *P < 0.05

The inflammatory consequences of TLR activation are well established for microglial cells, but largely unexplored for neuronal cells. Therefore, differentiated SHSY5Y cells were treated with a panel of MYD88-dependent TLR agonists and the expression of the pro-inflammatory cytokine, tumor necrosis factor α (TNFα), was measured. Results display a remarkable neuronal specificity for the TLR2 agonist (Fig. 4a), while all TLR agonists could induce TNFα expression in a macrophage cell line (Raw 264.7 cells) (Supplementary Figure 4). TLR2 is considered the most promiscuous of TLR receptors, being that it is able to bind a diverse selection of agonists including bacterial lipopeptides, yeast cell components and mycoplasma [43]. Thus, specificity was assessed using a panel of TLR2 agonists to treat SHSY5Y cells. The highest induction of TNFα was observed with the bacterial lipopeptide PAM3CSK4 (Fig. 4b), which specifically activates TLR2/TLR1 dimers [40]. Induction of TNFα was also seen with agonists that activate TLR2/TLR6 dimers (PAM2CSK4 and FSL1), while other agonists failed to elicit a response in SHSY5Y cells under the same conditions (Fig. 4b). Time course experiments showed that secretion of TNFα could be readily detected in tissue culture supernatant following treatment with PAM3CSK4 (Fig. 4c). Importantly, the induction of TNFα following PAM3CSK4 treatment was markedly blunted with a TLR2 neutralizing antibody, confirming specific activation of this TLR (Fig. 4d). In addition to the induction of TNFα, classical TLR2 signal transduction could be measured in SHSY5Y cells including activation of the mitogen-activated protein kinase (MAPK) pathway as evidenced by increased phosphorylation of p38 MAPK and the NFκB p105 subunit (Fig. 4e). Indeed, in addition to TNFα, other PD-implicated inflammatory cytokines, and in particular microglial recruiting and activating chemokines, were induced following neuronal TLR2 activation (Supplementary Table 1). Activation of TLR2 also induced oxidative stress in the SHSY5Y cells (Fig. 4f), but the levels of inflammatory cytokines or oxidative stress were insufficient to induce apoptosis or have an effect on cell viability (Fig. 4g). Also noted following TLR2 activation was a significant increase in the expression of TLR2 itself, both mRNA (Fig. 4h) and protein (Fig. 4i). The increase in TLR2 could be completely suppressed by cycloheximide (Fig. 4i) demonstrating that increased TLR2 protein is mediated by increased gene expression. In contrast, there was no difference in the mRNA expression of α-synuclein (Fig. 4j). We also saw no difference in the protein levels of TLR1 following PAM3CSK4 treatment of SHSY5Y cells (Supplementary Figure 5).

### Neuronal TLR2 activation increases α-synuclein protein in SHSY5Y cells

**Fig. 5:**
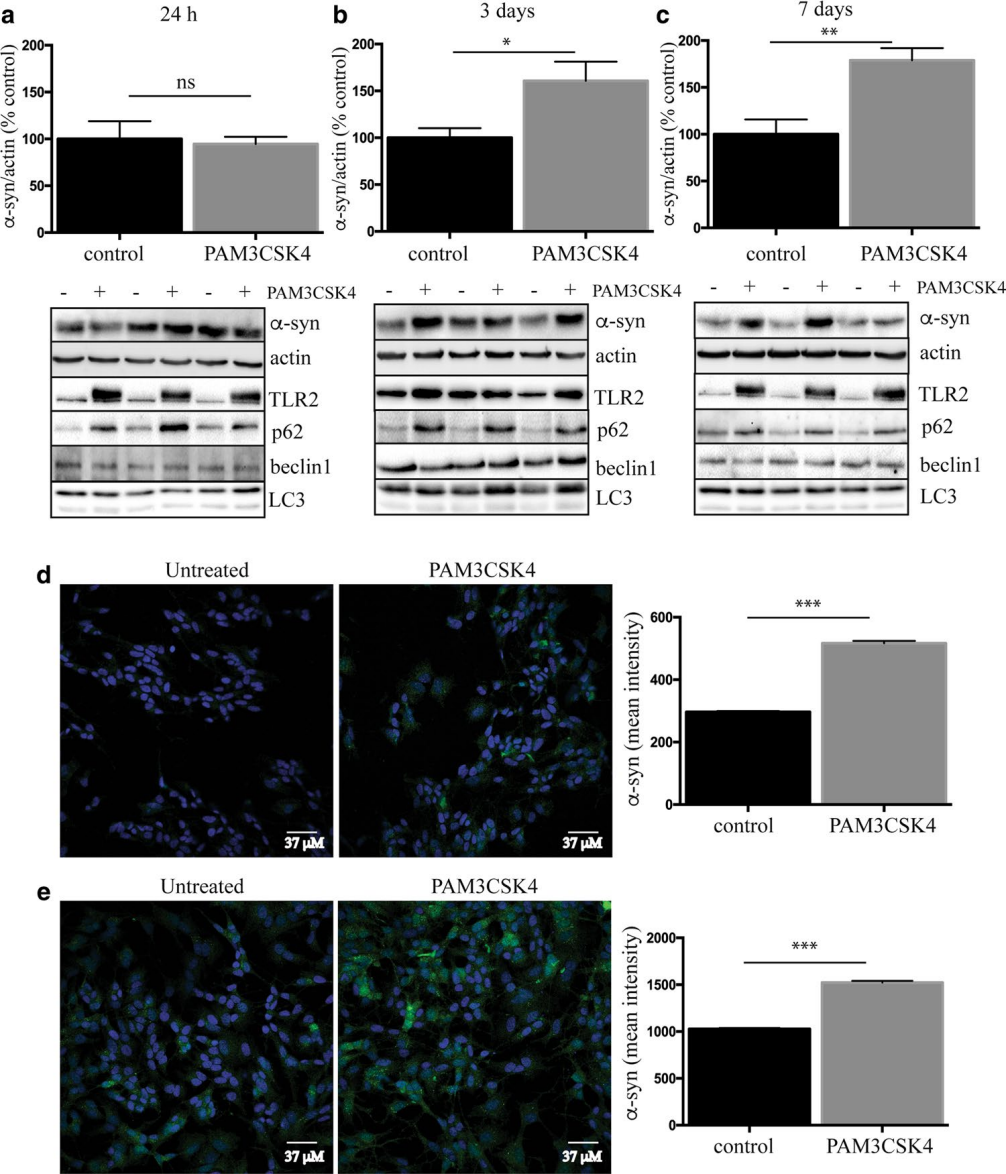
Activation of neuronal toll-like receptor 2 increases α-synuclein protein. Differentiated SHSY5Y cells were treated with 1 μg/ml PAM3CSK4 for **a** 24 h, **b** 3 days or **c** 7 days and cells collected into 1 × LDS sample buffer for immunoblot detection of α-synuclein, TLR2 and the autophagy proteins p62/SQSTM1, Beclin1 and LC3. For the 7 day treated cells, the media with or without 1 μg/ml PAM3CSK4 were replaced on day 3 and day 5. *Graphs* represent the quantified α-synuclein expression after correction to β-actin for loading. Data are expressed as mean ± SEM and are expressed as the percent increase over untreated cells, which were set to 100% and are based on *n* = 6–9. Representative immunoblots from at least three independent experiments are shown. Differentiated SHSY5Y cells were grown on coverslips and fixed after 3 (**d**) or 7 days (**e**) of 1 μg/ml PAM3CSK4 treatment for α-synuclein immunostaining (shown in *green*). The media were replaced on 7-day treated cells as above. Confocal images taken at ×40 magnification are representative of the 4–8 images containing 50–100 cells per image that were used for the analysis of staining intensity shown in the *graphs* as mean ± SEM. *P < 0.05, **P < 0.01, ***P < 0.001

As the human brain data showed a clear relationship between TLR2 and α-synuclein, we also measured α-synuclein protein levels in SHSY5Y cells treated with PAM3CSK4. After 24 h, levels of α-synuclein remained unchanged between control and TLR2-treated cells, despite the significant increase in TLR2 protein (Fig. 5a). By 3 days, however, significantly more α-synuclein could be measured in the PAM3CSK4-treated cells (Fig. 5b). Levels of α-synuclein were further increased again by 7 days (Fig. 5c). In contrast, 7-day treatment with the TLR4 agonist LPS failed to increase levels of α-synuclein protein (Supplementary Figure 6). As we did not see any effect of TLR2 activation on α-synuclein gene expression levels, and α-synuclein protein can be regulated by the lysosomal/autophagy pathway (for review see [46]), we measured key autophagy markers in the SHSY5Y cells. We saw no differences in beclin-1 levels or the conversion of LC3-I to LC3-II with TLR2 activation; however, a marked increase in p62 expression was observed (Fig. 5a–c) suggesting an impairment in selective lysosomal degradation pathways. Consistent with this concept was that treatment with the mTOR inhibitor and autophagy inducer, rapamycin, could prevent the accumulation of α-synuclein following TLR2 activation (Supplementary Figure 7). Also of note was that both TLR2 and p62 were increased with PAM3CSK4 treatment at 24 h, preceding the significant accumulation of α-synuclein. The effect of TLR2 activation on the accumulation of α-synuclein could also be readily observed using microscopy at both 3 (Fig. 5d) and 7 (Fig. 5e) days of treatment with PAM3CSK4.

### Neuronal TLR2 activation increases α-synuclein protein in primary human neural cells

**Fig. 6:**
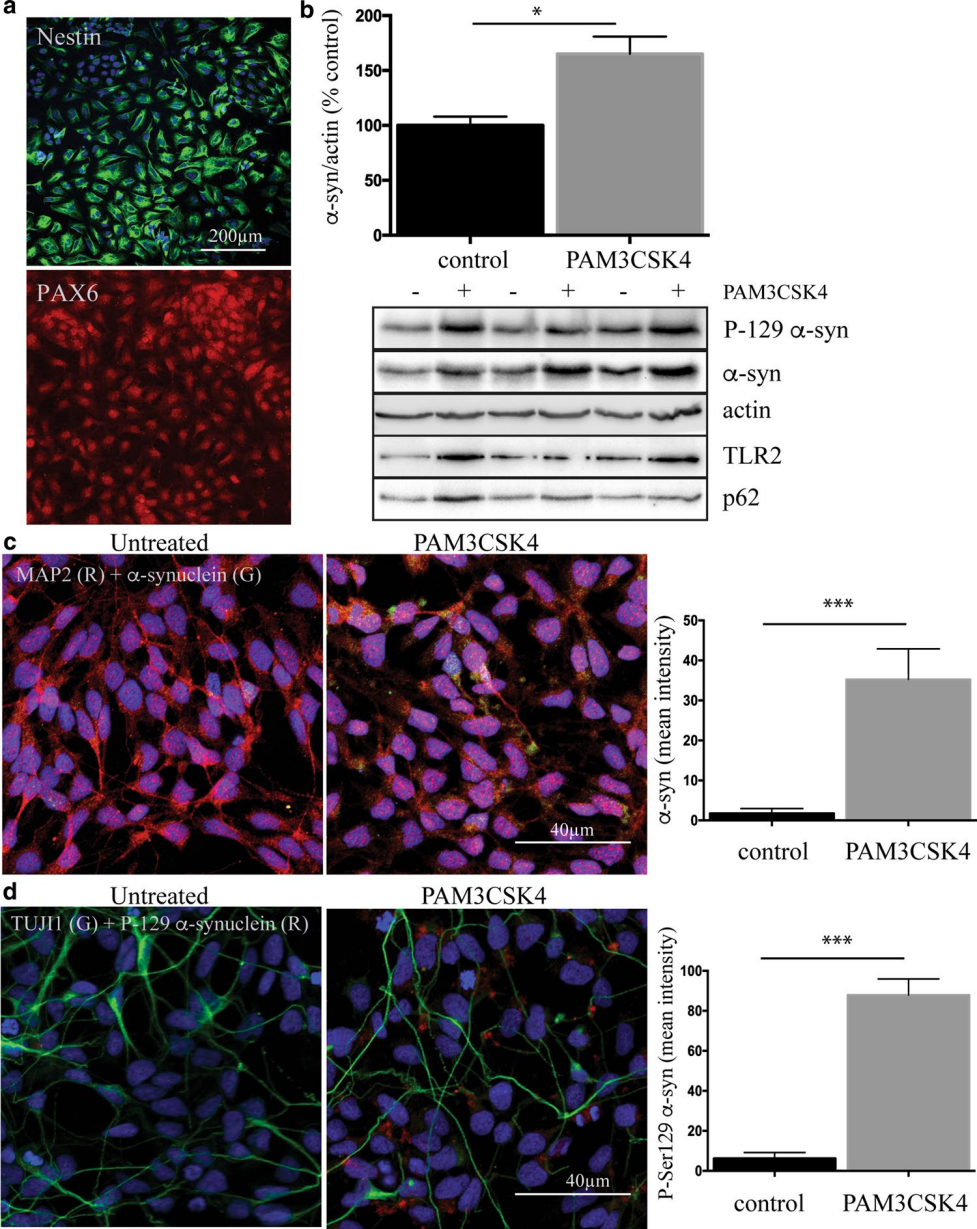
Activation of toll-like receptor 2 increases α-synuclein protein in IPS-derived neural cells. **a** Induced pluripotent stem cells were then differentiated into neural progenitor cells, which were strongly immunopositive for Nestin and PAX6. **b** Neural progenitor cells were then treated with 1 μg/ml PAM3CSK for 3 days and cells collected into 1 × LDS sample buffer for the immunoblot analysis of α-synuclein and TLR2. Representative immunoblots are shown from 2 independent experiments. *Graphs* represent the quantified α-synuclein expression after correction to β-actin for loading. Data are expressed as mean ± SEM and are expressed as the percent increase over untreated cells, which were set to 100% and are based on *n* = 6. *P < 0.05. Neural progenitor cells were plated on coverslips and further differentiated to MAP2 (*red* in **c**) and TUJ1 (*green* in **d**) positive neurons that were treated with 1 μg/ml PAM3CSK4 for 7 days. The media were replaced every second day and included fresh 1 μg/ml PAM3CSK4. Cells were then fixed and stained for α-synuclein (*green* in **c**) and P-ser129 α-synuclein (*red* in **d**). Confocal images are representative of the 5–7 images containing 20–40 cells per image that were used for the analysis of staining intensity shown in the *graphs* as mean ± SEM. ***P < 0.001. All cells in the images were used for the measurement of α-synuclein intensity

To confirm that the TLR2-mediated increase in α-synuclein could also be seen in primary human neural cells, we reprogrammed skin fibroblasts into induced pluripotent stem cells (Supplementary Figure 8) and then into neural progenitor cells. More than 95% of the progenitor cells expressed the neural stem cell markers nestin and PAX6 (Fig. 6a). We treated the progenitor cells with PAM3CSK4 for 3 days and again observed an increase in α-synuclein protein, as well as α-synuclein phosphorylated at the pathologically associated Ser129 residue (Fig. 6b). We then further differentiated the neural progenitor cells to neurons that no longer expressed nestin, but now did express the neural markers TUJ1 and MAP2 (Fig. 6c, d). Treatment of the induced pluripotent stem cell-derived neurons with PAM3CSK4 for 7 days again resulted in a marked increase in the immunostaining of both α-synuclein and α-synuclein phosphorylated at Ser129 (Fig. 6c, d). Thus, the results from both SHSY5Y cells and primary human neural cells support the clear relationship between TLR2 and α-synuclein protein seen in human postmortem PD brain.

### TLR2 signaling inhibitors prevent PAM3CSK4-mediated increases in α-synuclein protein in SHSY5Y cells

**Fig. 7:**
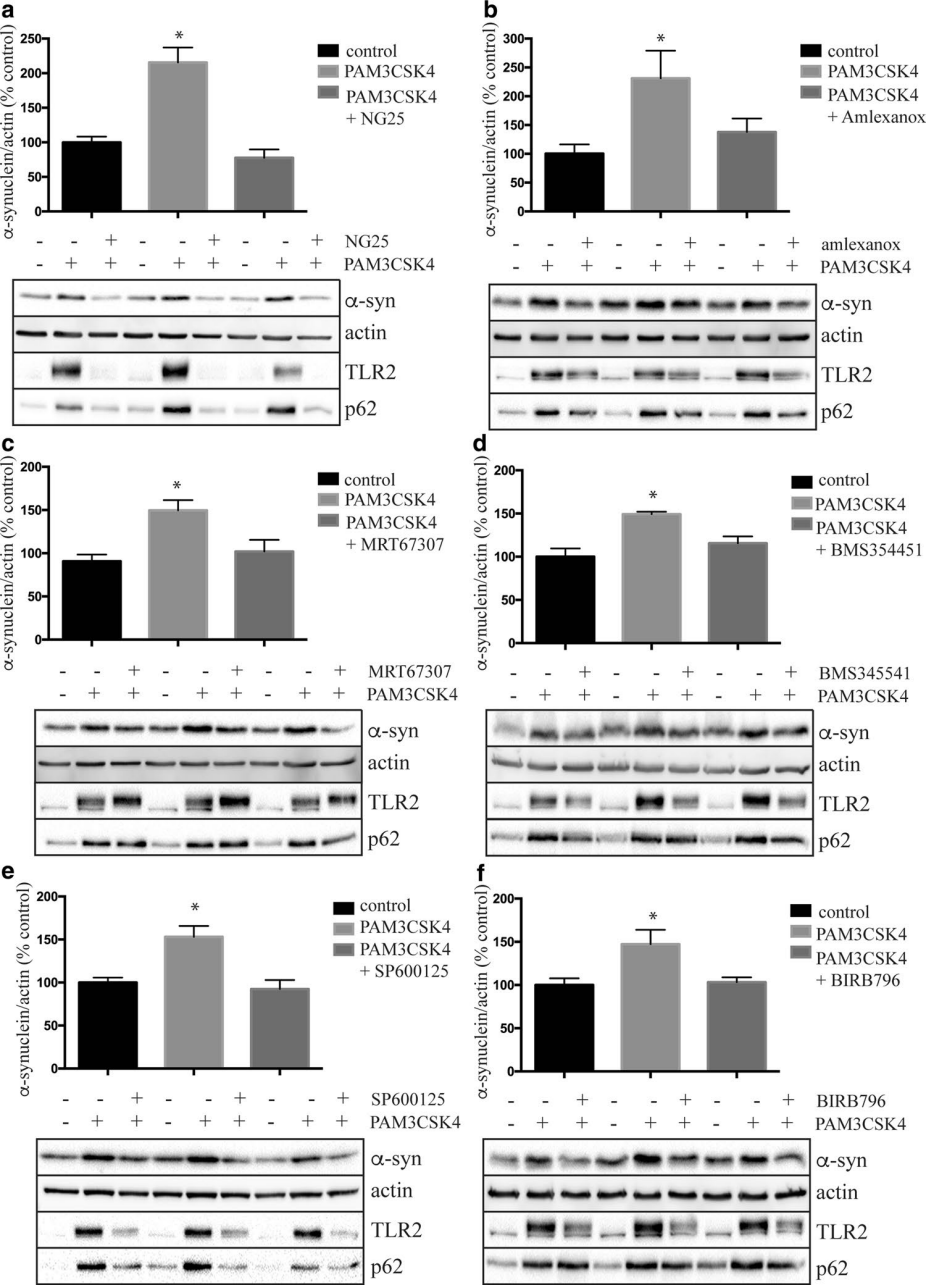
Toll-like receptor 2 signaling inhibitors can prevent the PAM3CSK4-mediated increase in α-synuclein protein. Differentiated SHSY5Y cells were treated with or without 1 μg/ml PAM3CSK4 in the presence or absence of 0.5 μM TAK1 inhibitor NG25 (**a**), 25 μM TBK1 inhibitor amlexanox (**b**), 2 μM TAK1 inhibitor MRT67307 (**c**), 2 μM IKK inhibitor BMS345541 (**d**), 5 μM JNK inhibitor SP600125 (**e**) and 2 μM p38 MAPK inhibitor BIRB796 (**f**) for 4 days. Concentrations of inhibitors were chosen based on initial dose–response experiments using SHSY5Y cells. The media were replaced every 48 h with new media containing fresh PAM3CSK4 and inhibitor. Cells were lysed directly into 1 × LDS sample buffer and immunoblotted for the indicated proteins. Representative immunoblots are shown and at least 2 independent experiments were performed in triplicate. Data are expressed as mean ± SEM and are expressed as the percent increase over untreated cells, which were set to 100% and are based on *n* = 6. *P < 0.05 compared to the untreated group

Finally, we employed a collection of small molecule inhibitors that target the TLR signal transduction pathway to determine if such compounds can prevent the accumulation of α-synuclein following TLR2 activation. The TAK1 inhibitor, NG25 (a kind gift from Nathanael Gray), clearly ameliorated the PAM3CSK4-mediated increase in both TLR2 and α-synuclein (Fig. 7a). Inhibition of the non-canonical IκB kinases, TBK1 and IKKε with amlexanox (Fig. 7b) and MRT67307 (Fig. 7c) could also ameliorate the TLR2-stimulated increase in α-synuclein, although only amlexanox also reduced TLR2. Inhibition of the IκB kinase beta (IKKβ) with BMS345541 also reduced both α-synuclein and TLR2 (Fig. 7d). Further downstream of the TLR2 pathway, inhibition of JNK (Fig. 7e) and p38 MAPK (Fig. 7f) with SP600125 and BIRB796, respectively, again ameliorated both the PAM3CSK4-stimulated increase in α-synuclein and TLR2 protein, consistent with the contribution of these transcription factors to the regulation of TLR2 gene expression following its activation. Although more work is required to determine the precise signaling events, these results suggest that targeting the TLR2 pathway may be a potential therapeutic option for preventing α-synuclein accumulation and/or preventing increases in TLR2.

## Discussion

Whether inflammation constitutes a cause or consequence of PD has been much debated; however, the increasing number of PD-associated genes being implicated in inflammatory pathways is suggestive of a causative role, at least in part [10, 12]. One such PD-associated gene is α-synuclein, which can cause autosomal dominantly inherited PD when mutated [34]. α-Synuclein is also the major component of PD-defining Lewy bodies [38], where it accumulates in higher molecular weight, hyperphosphorylated aggregates [14]. In brain, the innate immune response is perceived to be mediated by glial cells, and it has been demonstrated that α-synuclein can activate microglia [1, 3, 21, 39, 47]. Both oligomeric [19] and fibrilar [15] forms of α-synuclein have been shown to activate microglial TLR2 in tissue culture experiments. In addition, monomeric α-synuclein may potentiate inflammatory cytokine production by microglial cells stimulated with TLR agonists, in particular the prototypical TLR2 agonist PAM3CSK4 [35]. Finally, α-synuclein can increase the expression of TLRs on microglia [4], suggesting the potential for a detrimental feedback loop. Indeed, recent evidence suggests an upregulation of TLR2 on microglia in the substantia nigra of PD brain [11]. However, whether increased TLR2 is associated with α-synuclein pathology, which accumulates in neurons in human PD brain and, to what extent, has not been established.

To determine whether an association between TLR2 and α-synuclein pathology indeed occurs, we performed a detailed examination of the anterior cingulate cortex of patients with PD, a brain region with limited neuronal loss but a predilection for α-synuclein pathology. Compared to matched controls, a two-fold increase in TLR protein was found and this correlated significantly with levels of α-synuclein. TLR2 was detected on microglia, but also on neurons, with the number of neurons positive for TLR2 staining being significantly increased in PD brain (approximately 50% of neurons by late stage disease). In the anterior cingulate cortex, neuronal TLR2 expression increased with disease duration and disease stage. TLR2 immunoreactivity was also increased in the remaining dopaminergic neurons in the substantia nigra. Moreover, most α-synuclein-immunoreactive Lewy bodies had robust TLR2 immunoreactivity, confirming a strong association between TLR2 and α-synuclein pathology in PD brain. Our data also show that TLR expression occurs across the human brain in controls, mainly on microglia, but also at very low levels in neurons with regional disease-specific upregulation mainly in neurons. While a similar fold change in TLR2 protein expression has been shown in patients with dementia with Lewy bodies compared to controls [19], the association with increased neuronal expression has not been previously identified in patients with Lewy bodies. Increased TLR2 expression on enteric and hypothalamic neurons was recently reported in the context of obesity and peripheral changes [6, 37], further confirming the potential of this innate immune pathway in neuronal cells to respond to regionally relevant disease signals.

In addition to being expressed on neurons, TLR2 could also be activated in differentiated dopaminergic neuron-like SHSY5Y cells, primary human neural progenitor cells and more mature neurons. Activation of TLR2 with PAM3CSK4, an established and selective agonist of TLR1/2, resulted in the production of inflammatory cytokines such as TNFα and interleukin 1β, and other cytokines increased in the brains of patients with PD [27, 28, 30]. In addition to the production of pro-inflammatory cytokines, neuronal TLR2 activation also resulted in the robust production of microglial recruiting and activating chemokines, as well as mild oxidative stress. The intrinsic production of inflammatory cytokines and oxidative stress by neuronal SHSY5Y cells, however, was insufficient to promote cell death. These results suggest that activation of TLR2 on neurons in the short-term may contribute to the inflammatory milieu present in PD brain; however, reaching toxic levels of cytokines likely requires amplification of the inflammatory response following the recruitment and activation of microglia, as particularly occurs in the substantia nigra of PD brain.

In addition to establishing the association between TLR2 and α-synuclein in postmortem PD brain, we were also able to model aspects of this relationship in tissue culture. Activation of neuronal TLR2 resulted in a marked and rapid increase in both the mRNA and protein expression of TLR2. In contrast, increases in α-synuclein protein were only detected after ~72 h and occurred without changes in mRNA expression. This suggested a potential defect in the turnover of α-synuclein protein, which is not clearly understood but involves both proteosomal and lysosmal degradation [46]. We did not find differences in the autophagy markers beclin1 or LC3 following TLR2 activation; however, we did find that TLR2 activation increased the levels of p62/SQSTIM1, a receptor for selective autophagy of target proteins potentially including α-synuclein [22, 42, 45], that is known to increase when autophagy/lysosmal pathways are impaired. Interestingly, activation of autophagy with rapamycin was able to ameliorate the TLR2-mediated increase in α-synuclein, suggesting that activating autophagy can help clear α-synuclein protein, as has been seen before in animal studies [2, 23, 25, 32]. It is also important to note that while our manuscript was in preparation, Kim and colleagues reported similar findings regarding the activation of TLR2 on cell culture neurons and the accumulation of α-synuclein in association with increased p62 [20]. While there are some similarities in this aspect, it is noteworthy that our study employed only endogenous proteins while Kim et al. employed neuronal cells transduced with lenti-viral α-synuclein and treated with 10-fold higher amounts of PAM3CSK4. However, Kim et al., also extend their studies to animal models and show that α-synuclein-mediated neurodegeneration is attenuated by either knockout or knockdown of TLR2 in rodent PD models [20]. We have further added to this important proof of principal work by demonstrating that small molecule inhibitors targeting the TLR pathway can also ameliorate the TLR2-mediated increase in α-synuclein and, as such, TLR2 pathway inhibition could form the basis of new therapeutic interventions. Although, whether compounds specific to the TLR2 pathway will be required, and determining how such compounds work mechanistically by either reducing TLR2 protein levels and/or restoring autophagic flux will need to be determined. Most importantly, however, we provide data from human postmortem brain demonstrating that the relationship between TLR2 and α-synuclein is bona fide and that further translational efforts at targeting the TLR2 pathway are warranted.

## Electronic supplementary material

Below is the link to the electronic supplementary material.

Supplementary material 1 (PDF 7995 kb)
