# Supplementary material for: Toll-like receptor 2 is increased in neurons in Parkinson’s disease brain and may contribute to alpha-synuclein pathology
Source: Acta Neuropathol. 2016 Nov 25;133(2):303–19. doi: 10.1007/s00401-016-1648-8 (PMC5250664; doi:10.1007/s00401-016-1648-8)
Supplement: Supplementary file 1 — Supplementary material 1 (PDF 7995 kb) [file 401_2016_1648_MOESM1_ESM.pdf]

Toll-like receptor 2 is increased on neurons in Parkinson's disease brain and may contribute to alpha-synuclein pathology.

Nicolas Dzamko[1,2*], Amanda Gysbers[1], Gayathri Perera[1], Anita Bahar[1], Amrita Shankar[1,2], Jianqun Gao[1,2], YuHong Fu[1,2] and Glenda M Halliday[1,2*]

[1]Neuroscience Research Australia, Randwick, NSW, 2031, Australia

[2]School of Medical Sciences, University of NSW, Sydney, NSW, 2052, Australia

***Correspondence to**: Dr Nicolas Dzamko n.dzamko@neura.edu.au or Professor Glenda Halliday g.halliday@neura.edu.au, Neuroscience Research Australia, Barker Street, Randwick, NSW 2031, Australia

SUPPLEMENTARY METHODS

Immunoperoxidase staining with anti-TLR2 (abcam, 108998)

Sections were incubated with 5% H_2O_2 in 50% ethanol for 30 min before blocking with 10% horse serum in TBS buffer containing 0.5% Triton X-100. Sections were then incubated overnight at 4 °C with anti-TLR2 (1:100 dilution) or anti-α-synuclein (BD, 1:200 dilution) prepared in 1% horse serum. Biotinylated secondary antibody (Vector Laboratories) was used for 30 min at 37 °C followed by incubation in avidin-biotin complex (ABC Vectastain kit, Vector laboratories) for 30 min at room temperature. Peroxidase immunolabelling was visualized with nickel enhanced 3'3' diaminobenzidine tetrahydrocholoride (DAB) solution (Sigma) containing 0.5% H_2O_2, and sections were counterstained with cresyl violet acetate (buffered 0.5%, Sigma), washed in 0.1 M Tris buffer, dehydrated through graded ethanols to xylene and coverslipped using Di-n-butyl phthalate in xylene (DPX) (Sigma). Images to assess the localization of these proteins were visualised using a Zeiss Axioskop 2 microscope and captured with a Zeiss AxioCam HRc camera and Axiovision 4.2 software.

Immunoperoxidase staining with anti-TLR2 (R&D systems, AF2616)

For this TLR2 antibody (R&D Systems, AF2616), Tris-EDTA (pH9.0) was used for antigen retrieval. Sections were incubated with 0.3% H_2O_2 and 0.1% sodium azide in TBS for 20 min to block endogenous peroxidase activity, before blocking with 10% horse serum in TBS buffer containing 0.5% Triton. Sections were incubated overnight at 4 °C with anti-TLR2 (1:1000 dilution) prepared in 4% horse serum. Sections were then washed and incubated in biotinylated secondary antibody (Vector Laboratories) for 2 hours at room temperature followed by incubation in avidin-biotin complex (ABC Vectastain kit, Vector laboratories) for 1 hour at room temperature. Peroxidase immunolabeling and imaging were carried out as above.

Antibody pre-absorption

Antigen (Recombinant human TLR2, R&D Systems, AF2616-TR) to antibody mixture were made at a working dilution of 10:1 (molar ratio) and were incubated overnight 4 °C. The pre-absorbed antibody complexes were then incubated with tissue sections in place of the primary antibodies as outlined above.

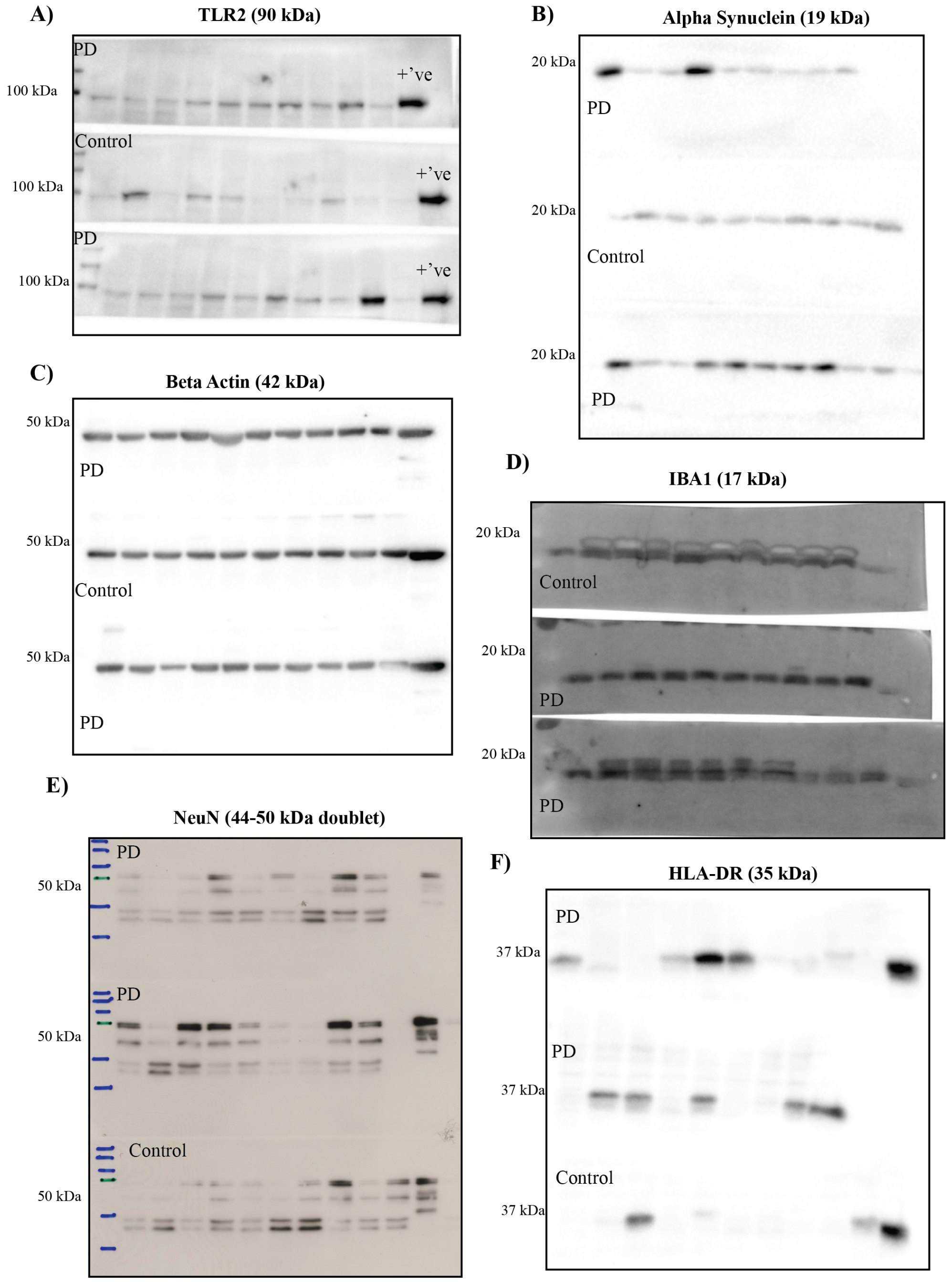

Supplementary Figure 1. Uncropped immunoblot images used for quantitation of TLR2 and α-synuclein in human brain. 20-30 μg of SDS-soluble protein lysate was resolved on 4-12% gradient gels and transferred to nitrocellulose membrane. Membranes were cut into strips to allow immunoblot of different size proteins. The number of cases dictated that three strips were required for each protein of interest. Membrane strips were placed in strip containers and processed identically. All three strips were then visualized simultaneously using a Chemidoc MP Imager. Molecular weight markers were also recorded. Human peripheral blood mononuclear cells (PBMCs) were used as a positive control for expression of TLR2.

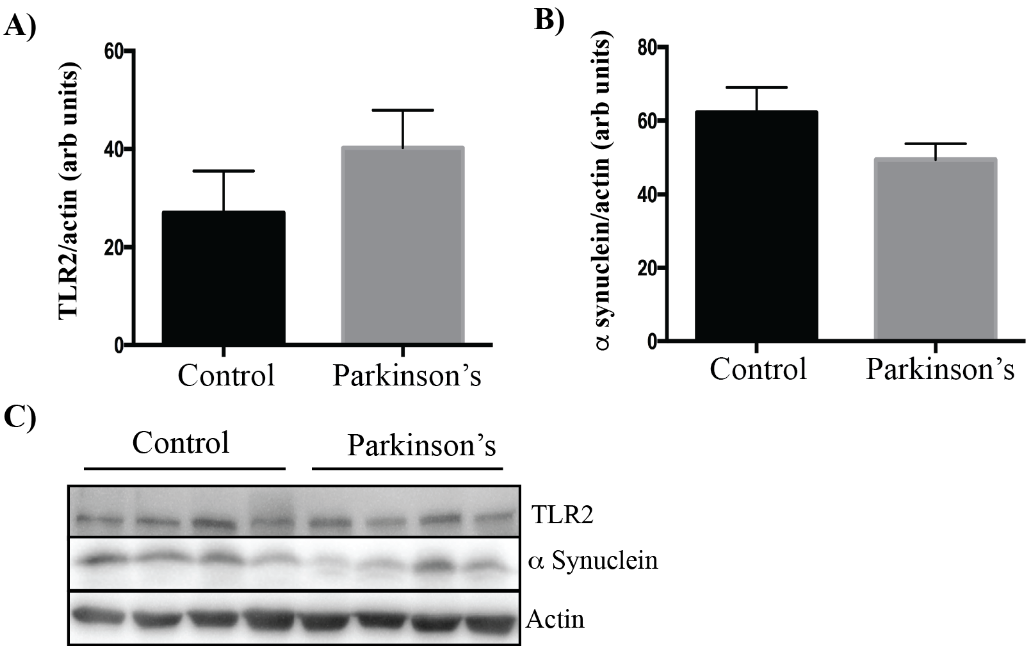

Supplementary Figure 2. TLR2 is not increased in occipital cortex of Parkinson's disease brain. Quantification of TLR2 **(a)** and SDS-soluble total α-synuclein **(b)** protein in control and Parkinson's disease (PD) occipital cortex after normalization to β-actin. Data are mean ± SEM, n = 10 controls and 17 PD cases. **c**) Representative immunoblot images from control and PD occipital cortex.

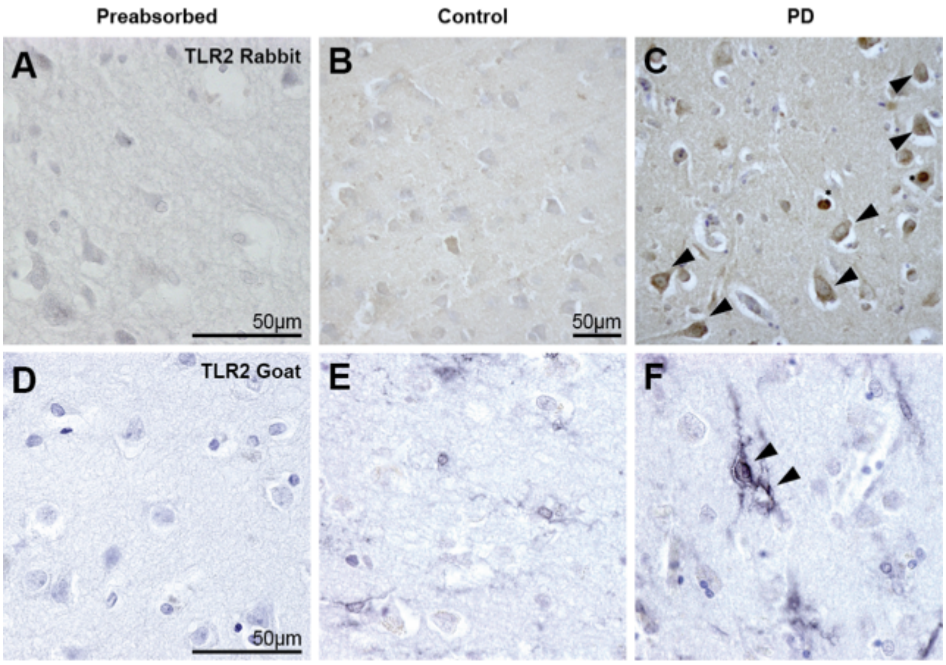

Supplementary Figure 3. Increased toll-like receptor 2 protein is expressed in neurons in Parkinson's disease brain. Representative peroxidase immunostaining of TLR2 in sections of the anterior cingulate cortex using two different antibodies (anti-rabbit TLR2 (Abcam, 108998) (**a-c**) and anti-goat TLR2 (R&D Systems, AF2616) (**d-f**) as outlined in the supplementary methods. Cells of neuronal morphology (indicated by arrows) are only observed as TLR2-immunopositive in PD **(c, f)** compared with controls **(b, e)**. Microglial processes can be seen at higher magnification in both controls and PD cases (**e, f**) but neuronal TLR2 immunopositivity is only observed in PD (**c, f**) using these methods. Preabsorption of both TLR2 antibodies with recombinant human TLR2 abolished positive staining (**a, d**). Scale in **b** is equivalent for **c**. Scale in **d** is equivalent for **e** and **f**.

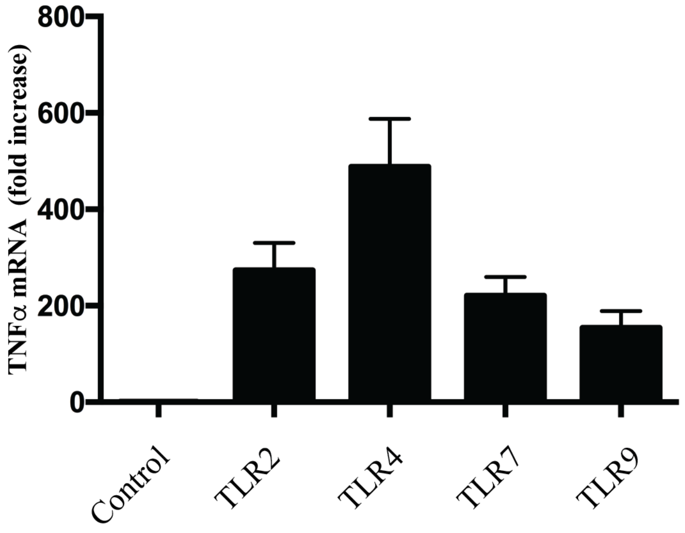

Supplementary Figure 4. MYD88-dependent TLR agonists induce TNFα expression in macrophages. Murine RAW 264.7 cells were treated with agonists of toll-like receptor signaling for 6 h before RNA was extracted for measurement of TNFα mRNA expression by qRT-PCR (TLR2 agonist = 1 μg/ml PAM3CSK4, TLR4 agonist = 1 μg/ml lipopolysaccharide, TLR7 agonist = 1 μg/ml CLO97 and TLR9 agonist = 2 μM ODN 2336). Mouse specific TNFα primers were used. Data are mean ± SEM following normalization to GAPDH, n = 6.

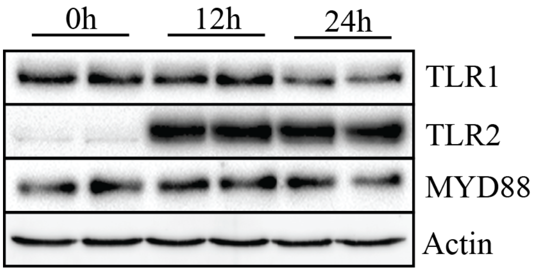

Supplementary Figure 5. Activation of TLR2 does not increase TLR1 protein in SHSY5Y cells. Differentiated SHSY5Y cells were treated with 1 µg/ml PAM3CSK4 for either 0, 12 or 24 hours. Cells were lysed directly into 1 x LDS sample buffer and immunoblotted for the indicated proteins. Representative immunoblots are shown and 3 independent experiments were performed.

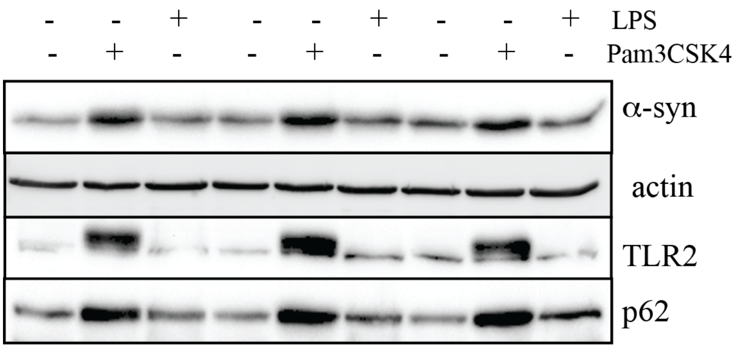

Supplementary Figure 6. LPS treatment fails to increase α-synuclein levels. Differentiated SHSY5Y cells were treated with or without 1 µg/ml PAM3CSK4 or 1 µg/ml LPS for 7 days. The media was replaced every 48 h. Cells were lysed directly into 1 x LDS sample buffer and immunoblotted for the indicated proteins. Representative immunoblots are shown and 3 independent experiments were performed.

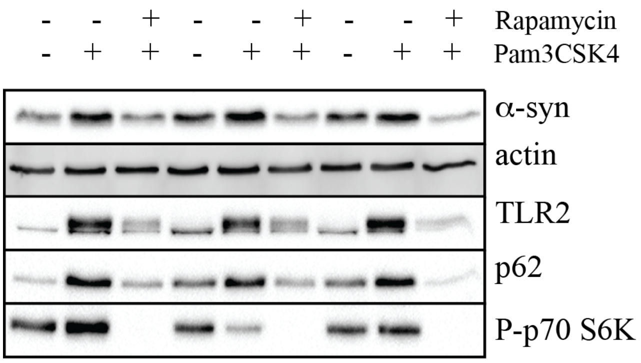

Supplementary Figure 7. Rapamycin prevents the TLR2-mediated increase in α-synuclein. Differentiated SHSY5Y cells were treated with or without 1 μg/ml PAM3CSK4 for 4 days in the presence/absence of 5 μM rapamycin. The media was replaced every 48 h. Cells were lysed directly into 1 x LDS sample buffer and immunoblotted for the indicated proteins. Representative immunoblots are shown and 3 independent experiments were performed.

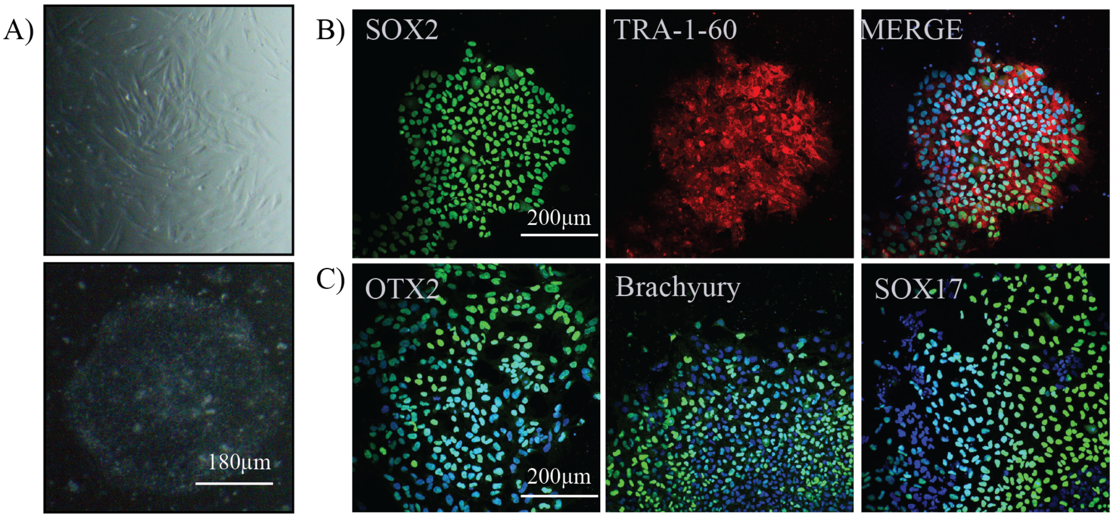

Supplementary Figure 8. Generation of induced pluripotent stem cells. a) Fibroblasts were electroporated with epi5 reprogramming vectors and colonies with induced pluripotent stem cell morphology were isolated after 30-40 days. **b)** The clone used for this study stained highly positive for the pluripotency markers SOX2 and TRA-1-60 and was capable of differentiation into the three germ layers as evidenced by positive staining for OTX2 (ectoderm marker), Brachyury (mesoderm marker) and SOX17 (endoderm marker) (**c**).

Cytokine	Concentration (pg/ml) ± SEM	Increase (%)	Significance
IL-8	46.4 ± 11.1	1950	***
CXCL10	82.8 ± 15.8	1646	***
CCL5	116.2 ± 19.0	3632	***
G-CSF	36.2 ± 2.4	3156	***
MCP-1	665.9 ± 37.5	506	***
TNF- α	11.7 ± 1.2	179	***
IL-15	7.2 ± 0.7	164	**
IL-1β	0.5 ± 0.0	37	**
IL-7	3.4 ± 0.1	40	**
CCL4	1.1 ± 0.1	79	**
IL-12	174.7 ± 10.8	73	**
IL-13	4.5 ± 0.2	52	**
IFN-γ	14.8 ± 0.7	61	*
IL-10	26.5 ± 2.4	55	*
IL-1Ra	17.3 ± 1.2	53	*
IL-17A	7.5 ± 0.7	89	*
GM-CSF	206.4 ± 6.4	104	*
IL-4	0.4 ± 0.0	36	
Eotaxin	4.2 ± 0.0	27	
IL-9	3.4 ± 0.5	6	
IL-2	2.0 ± 0.2	49	

Supplementary Table 1. Inflammatory cytokines and chemokines expressed by SHSY5Y cells following TLR2 activation. Differentiated SHSY5Y cells were stimulated with 1 µg/ml PAM3CSK4 for 12 h and tissue culture media removed for the assay of inflammatory

cytokines and chemokines using a multi-plex ELISA. Concentrations were determined using a 5-paramater logarithmic standard curve and the percent increase measured compared to untreated cells. Significance was assessed by t-test with * = $P < 0.05$, ** = $P < 0.01$ and *** = $P < 0.001$. n=6. CCL4: Chemokine C-C motif ligand 4; CCL5: Chemokine C-C motif ligand 5; CXCL10: Chemokine C-X-C motif ligand 10; G-CSF: Granulocyte colony-stimulating factor; GM-CSF: Granulocyte Macrophage Colony-Stimulating Factor; IFN-γ: Interferon gamma; IL-1β: Interleukin-1 beta; IL-1Ra: Interleukin-1 receptor antagonist; IL-2: Interleukin 2; IL-7: Interleukin 7; IL-8: Interleukin 8; IL-9: Interleukin 9; IL-10: Interleukin 10; IL-12: Interleukin 12; IL-13: Interleukin 13; IL-15: Interleukin 15; IL-17A: Interleukin 17A; MCP-1: Monocyte Chemoattractant Protein-1; TNF- α: Tumor necrosis factor-alpha;
